# 

**Published:** 1889-04

**Authors:** 


### TABLE OF CONTENTS.

ORIGINAL AND SELECTED.

Address on the Divine Mission of the Medical Profession........ 121
Origin ot the Diphtheretic Virus, Dr. Deichler. 130
A Case of Hepatic Stone, &c., E. T. Saunders, M. D., ........ 134
The Treatment of Diphtheria. ........ 135

ABSTRACTS &c.

Gonorrhœa in the Female. ........ 136
Ventro Fixation in Retroflexion........ 137
Antipyrin and its Substitutes, &c., Papein, &c. 138
Dyspeptic Dyspnœa. ........ 139
A Case of Sciatica, &c., Pleurodynia, &c. ..... 140
Pneumonia &c,, On the administration of Pichi, &c. ........ 141
Treatment of Erysipelas, &c, Treatment of Deafness, &c., Graves' Disease &c. ........ 142
Scarlantina and Bronchitis, &c., Brief outline of Teachings of Surgery, &c. ........ 143
Magisterium Bismuthi, &c,, To Blister the Skin Quickly, &c., Thomas Oliver, M. D., says ot Adonidine:, &c. ........ 144
For Gastro-intestinal Catarrh, &c., Boiled water &c, Epistaxis from Salicylism, &c ........ 145
Antipyrin in Laryngismus, &c., Dangerous Antipyrin, &c., Sir Morrell Mackenzie, &c., In a Communication, &c.. ........ 146
The Antilithic Properties, &c., Juniper Berries, &c., Antipyrin in Obstetric Practice, &c....... 147
Chorea, &c., Electric Illumination, &c.. ........ 148
Coryza, &c., Dangers of Sulphonal, &c., Dr. A. H. Newth, &c ........ 149
Monthly Summary, &c., In La Progres Medical, &c. ........ 150

SCIENTIFIC

Glacier studies, &c., Californian big trees, &c., Securite, &c., Electric current speed, &c., All the land &c., The Canals of Mars, &c. ..... 151
Sun spots, &c., Easy experiment, &c., Saccharine Prohibited, &c. ........ 152

NOTES AND FORMULÆ.

Rheumatism, &c., Lithium and Arsenic, &c., Cancer, &c., New Preserving Fluid, &c. ..... 153
For Ringworm, &c., Chilblains, &c., Pomade for Antiseptic Dressing, &c., Eczema Marginatum, &c., Albuminaria of Pregnancy, &c., Chronic Interstitial Nephritis, &c.. ........ 154

EDITORIAL.

The Editor Burnt out, &c., Action Taken by his Colleagues, &c. ........ 155
So. Med. College Commencement, &c. ........ 156
Medical Association of Georgia, &c., Atlanta Medical College Commencement, &c. ..... 158
Dr. John B. Hamilton resigns, &c........ 159
Special Notes. ........159 160

